# Virtual Interaction to Promote Mental Health in Children with Social Anxiety Disorders (VISAKI)

**DOI:** 10.1192/j.eurpsy.2026.11146

**Published:** 2026-07-31

**Authors:** A. Kretschmer-Trendowicz, F. Moser, L. Gürster, C. Pippirs, M. Steininger, P. Maas, A. Zeiler, S. Walk, C. von Bock, B. Krüger, T. Spittler

**Affiliations:** 1 Clinic for Child and Adolescent Psychiatry, Bezirkskrankenhaus Landshut, Landshut; 2 Deggendorf Institute of Technology, Deggendorf; 3 Department of Epileptology, University Hospital Bonn, Bonn; 4 Youse GmbH, Berlin; 5 Solid White, Stuttgart; 6 University of Bonn, Bonn, Germany

## Abstract

**Introduction:**

Social Anxiety Disorder (SAD) is on of the most prevalent psychiatric disorders in childhood, significantly affecting social development and personality. Symptoms include intense fear of negative evaluation, avoidance of social interactions, and somatic complaints such as palpitations, trembling, and sweating. If left untreated, SAD often persists into adulthood and increases the risk for conditions such as depression or substance abuse. Early detection and treatment are therefore crucial. Research shows that Cognitive Behavioral Therapy (CBT) effectively reduces symptoms and improves children’s quality of life and social functioning. However, long-term therapeutic success depends on the transfer of learned skills into everyday life. Supporting this transfer is especially challenging for children with limited access to psychotherapy. Virtual solutions may offer an opportunity to facilitate this critical phase and ensure therapeutic success.

**Objectives:**

This project aims to develop and implement a virtual reality (VR) platform that enables children to practice social skills in a controlled environment and to engage in social situations of increasing difficulty. The platform is designed to bridge the gap between therapeutic setting and children’s everyday social environments. It also allows for repeated practice across therapeutic settings, potentially enhancing outcomes. Core research questions include: How should virtual environments and avatars be designed to be therapeutically effective? Do gamification-based approaches improve acceptance and motivation in children? In what ways can multi-user approaches be ethically justified, therapeutically beneficial, and appropriately applied when working with highly vulnerable populations?

**Methods:**

The VR platform targets children aged 8–12 years. It will feature a real-time, three-dimensional system simulating complex social situations. A modular design and interactive gamification elements will encourage collaboration and practical social skill application. Scenarios will be presented using VR headsets. Pre- and post-intervention assessments will evaluate changes in social anxiety levels and the platform’s effectiveness in reducing symptoms.

**Results:**

The platform is currently under development. Children, parents, and therapists are being interviewed to design an interface that meets the needs of all stakeholder groups. The latest version of the platform, along with a detailed description of the methodological and technical aspects, will be presented at the conference.

**Conclusions:**

Initial conclusions from the research project will be presented at the conference.

**Disclosure of Interest:**

None Declared

